# Supplementary material for: Endosonography-Guided Versus Percutaneous Gallbladder Drainage Versus Cholecystectomy in Fragile Patients with Acute Cholecystitis—A High-Volume Center Study
Source: Medicina (Kaunas). 2022 Nov 14;58(11):1647. doi: 10.3390/medicina58111647 (PMC9699066; doi:10.3390/medicina58111647)
Supplement: Supplementary file 1 [file medicina-58-01647-s001.zip › medicina-1977629-supplementary.pdf]

Supplementary Table S1. Adverse events (Complications) and Severe Adverse Events (CD>3).

	**Overall**	**PT-GBD**	**EUS-GBD**	**LC**	**OC**	***p***
Overall complication (%)	41 (25.6)	13 (39.4)	2 (9.5)	13 (16.0)	13 (52.0)	**<0.001**
Clavien Dindo >3 (%)	16 (10.0)	9 (27.3)	1 (4.8)	2 (2.5)	4 (16.0)	**0.001**
Clavien Dindo I- V (%)						**<0.001**
0	126(78.8)	20 (60.6)	20 (95.2)	73 (90.1)	13(52.0)	
I	6 (3.8)	4 (12.1)	0 (0.0)	1 (1.2)	1 (4.0)	
II	12 (7.5)	0 (0.0)	0 (0.0)	5 (6.2)	7 (28.0)	
IIIa	6 (3.8)	5 (15.2)	0 (0.0)	0 (0.0)	1 (4.0)	
IIIb	4 (2.5)	3 (9.1)	0 (0.0)	0 (0.0)	1 (4.0)	
IVa	2 (1.2)	0 (0.0)	0 (0.0)	0 (0.0)	2 (8.0)	
IVb	1 (0.6)	0 (0.0)	1 (4.8)	0 (0.0)	0 (0.0)	
V	3 (1.9)	1 (3.0)	0 (0.0)	2 (2.5)	0 (0.0)	
